# Radiologic changes of long term foot insole use in symptomatic pediatric flatfoot

**DOI:** 10.1097/MD.0000000000033152

**Published:** 2023-03-10

**Authors:** Joon Yeop Kim, Soo A Kim, Yuntae Kim, Insu Hwang, Nam Hun Heo

**Affiliations:** a Department of Physical Medicine and Rehabilitation, Soonchunhyang University Cheonan Hospital, Soonchunhyang University College of Medicine, Cheonan, Republic of Korea; b Clinical Trial Center, Soonchunhyang University Cheonan Hospital, Cheonan, Republic of Korea.

**Keywords:** foot insole, pediatric, radiology, symptomatic flexible flatfoot

## Abstract

Clinically, flatfoot, known as pes planus, is quite prevalent. It is classified into 2 types: flexible and rigid, both of which may or may not have symptoms. If a flexible flatfoot is symptomatic, it must be treated to prevent subsequent complications. In principle, most physicians initially use conservative methods, such as foot insoles. This study aimed to demonstrate the effect of long term use of a foot insole using plain radiography as an objective measurement in children with symptomatic flexible flatfoot (SFFF) in large samples. This study analyzed the medical records of 292 children aged < 18 years who were diagnosed with SFFF. Of these, 200 children (62 boys and 138 girls, mean age: 6.49 ± 2.96 years) were selected and conservatively treated with foot insoles. They were periodically followed up within 3 to 4 months to modify the foot insole and perform radiologic evaluations, such as foot radiography. The calcaneal pitch angle (CPA) and talo first metatarsal angle were measured and compared individually using foot lateral radiographs, which were pictured in a bilateral barefoot state. The treatment was terminated by repeating the same procedure until the symptoms disappeared. A significant improvement (*P* < .001) was observed in the radiological indicators, both CPA and talo first metatarsal angle, regardless of age, after the application of soft foot insoles. However, the right foot CPA in the group with valgus deformity was an exception (*P* = .078). This study showed that in children diagnosed with SFFF under 18 years of age, wearing a periodically revised foot insole as conservative treatment could not only decrease the symptoms, but also improve the radiologic indices.

## 1. Introduction

Flatfoot, known as pes planus, is traditionally defined as any condition of the foot that results in loss or a lowered medial longitudinal arch than normal parameters, with the hindfoot in excessive valgus alignment.^[1–3]^ The true incidence of flatfoot is unclear because there is no consensus on clinical or radiographic definitions.^[2,4]^ One study showed flatfoot in 97% of 18-month-old and only 4% of 10-year-old,^[5]^ while another study revealed flatfoot in 54% of 3-year-old and 26% of 6-year-old.^[6]^ Both studies supported the idea that the majority of pediatric flatfoot cases resolve spontaneously without treatment.

Empirically, flatfeet are classified into 2 types: flexible and rigid. Flexible flatfoot shows a lowered medial longitudinal arch (LMLA) with calcaneal valgus in a weight-bearing position, but returns to its original shape in a non-weight-bearing position and is commonly asymptomatic. However, rigid flatfoot shows significant restriction of the subtalar joint motion and is usually symptomatic. In addition, it generally presents with comorbidities, such as coalitions of the foot bones and genetic syndromes.^[1,7,8]^ Symptoms related to flatfoot include pain in any part of the lower extremity, voluntary withdrawal while performing physical activities, and deformity of the foot bone.^[8]^ Most deformities are physiological. However, severe deformities can lead to various complications, such as pelvic alignment syndrome, shortening of the tendon, and poor quality of life.^[9,10]^ To prevent complications and improve the patient’s quality of life, interventions are needed to determine whether the treatment should be conservative or involve surgical procedures.^[8,11,12]^ In pediatric flexible flatfoot, foot orthosis is universally used as a first line conservative treatment.^[13]^

Overall, some previous studies have shown positive effects of conservative treatment for pediatric flexible flatfoot.^[4]^ However, it is difficult to gather diverse results owing to the different durations of the research and the variety of tools for conservative treatments. We collected all the data of periodic outpatient follow up until the symptoms disappeared, and the conservative treatment method was unified into 1 type of foot insole. The hypothesis is that the long term use of foot insoles in children would change the structure of the foot. This study aimed to prove the effectiveness of long term use of a foot insole by using radiography as an objective measurement of symptomatic flexible flatfoot (SFFF) in children in large samples.

## 2. Methods

### 2.1. Patient selection

**Figure 1.**
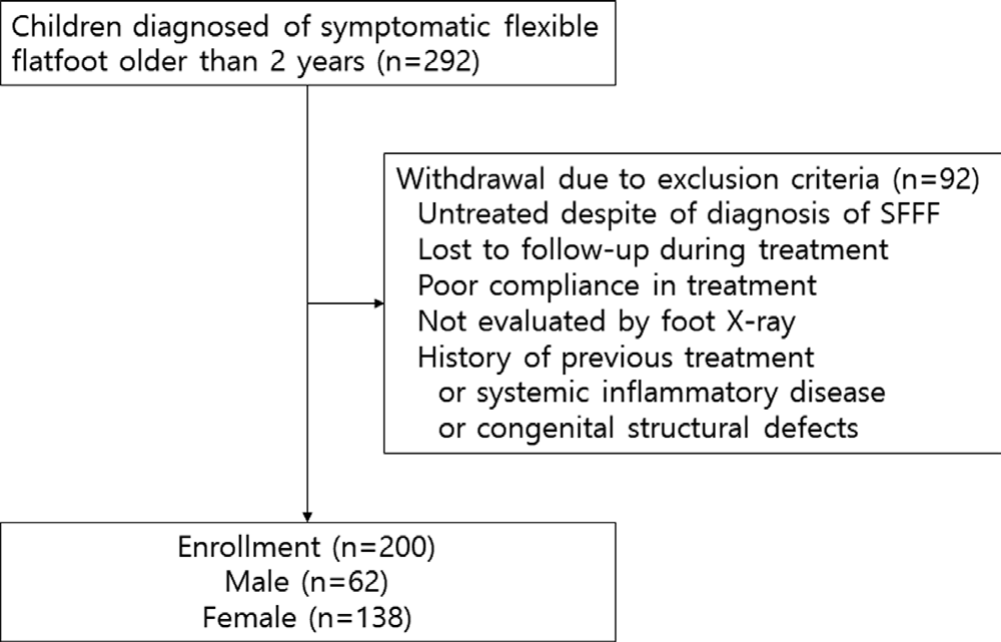
Flow chart of study selection.

A total of 292 participants under 18 years of age and diagnosed with SFFF at the Department of Rehabilitation Medicine, OOO Hospital were retrieved retrospectively from a hospital database system for managing outpatients. They were treated and followed up for a minimum of 1 year and a maximum of 6 years, periodically for 3 to 4 months through the outpatient clinic between November 2016 and April 2022. The following exclusion criteria were applied: Untreated despite a diagnosis of SFFF; Lost to follow up during the treatment; Poor compliance with the treatment; Not evaluated by foot radiographs, and; Having a history of previous treatment, systemic inflammatory disease, or congenital structural defects that could affect lower extremity alignment (Fig. 1). After exclusion, children were treated with foot insoles. This study was approved by the Institutional Review Board of OOO Hospital, Korea (No 2022-01-018). The requirement for informed consent was waived owing to the retrospective chart review.

### 2.2. Data sources and radiographic measurement

**Figure 2.**
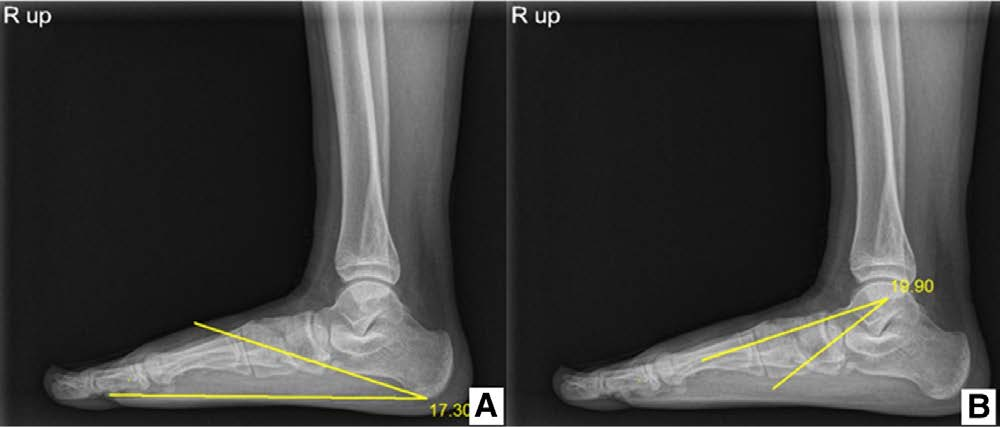
(A) Calcaneal pitch angle (CPA) and (B) Meary angle (talo-first metatarsal angle) (TMA) by standing lateral radiographs of foot. CPA = calcaneal pitch angle, TMA = talo first metatarsal angle.

The general characteristics of the enrolled subjects included age, sex, and flexible flatfoot conditions. Both lateral foot radiographs of the children at the time of diagnosis and at the end of treatment were taken to evaluate the effects of foot insole application in a barefoot standing position. Foot lateral radiography was used for radiographic measurement. The bilateral calcaneal pitch angle (CPA) and Meary’s angle, known as the talo first metatarsal angle (TMA), were measured in both feet. CPA is defined as the angle between the calcaneus and inferior aspect of the foot. TMA is defined as the angle between the line of the longitudinally bisected talus and the longitudinal axis of the 1^st^ metatarsal bone (Fig. 2). Both radiologic indices calculated through foot lateral radiography in a standing position are usually used clinically as criteria for flexible flatfoot: CPA < 15’; TMA > 3’.^[14]^ Both indices were periodically followed up within 3 to 4 months after the beginning of the foot insole prescription. The process of radiologic evaluation with adjustment of the foot insole was terminated at the point of loss of the associated symptoms, as mentioned above. All the radiographic parameters were measured by a trained physiatrist.

**Figure 3.**
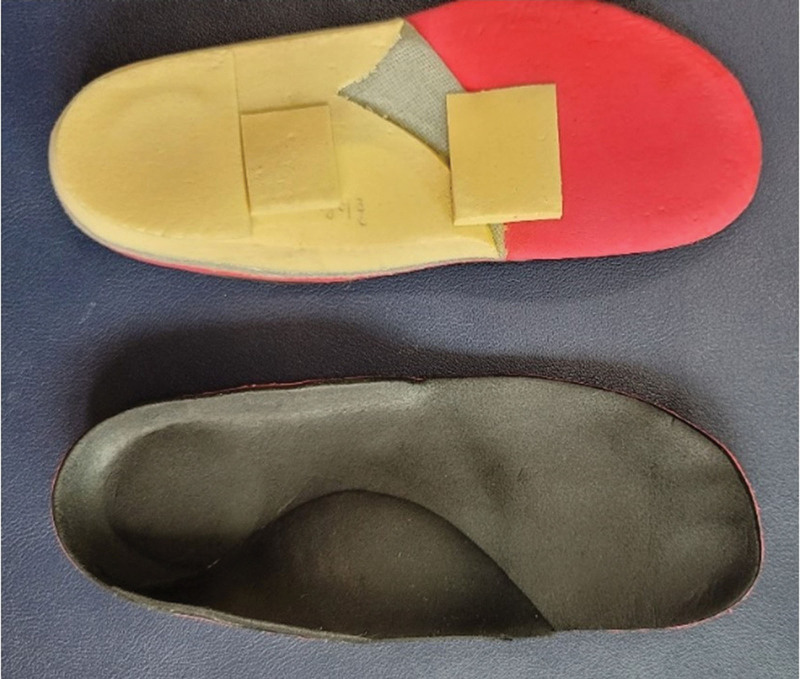
The foot insole used in this study.

The foot insole was also adjusted periodically for 3 to 4 months after confirmation of the follow up radiograph. The device was custom-made using ethylene vinyl acetate with foam materials. This supported the medial longitudinal arch (Fig. 3). During the intervention, the foot insole was revised according to the height of the pad.

### 2.3. Statistical analysis

Statistical analysis of the data was performed using SPSS 26.0 for Windows (SPSS Inc., Chicago, IL). Categorical variables are expressed as counts and percentages. Continuous variables were expressed as mean ± standard deviation. Categorical variables were analyzed using the chi-squared test or Fisher exact test. The paired *T* test or Wilcoxon log rank test was used to compare the change in both feet in CPA and TMA before and after treatment. Statistical significance was defined as a *P* value <.05 for all analyses.

## 3. Results

Of the 292 children with SFFF enrolled for the analysis, 200 children were completely followed up, and the foot insole treatment was terminated. The boy-to-girl ratio was 62 to 138. Their average age was 6.49 ± 2.96 years (Table 1). The children were divided into subgroups based on the characteristics of valgus deformity and LMLA. There was no difference between the valgus deformity alone group and the valgus deformity in the LMLA group (*P* = .669), regardless of age (*P* = .675).

**Table 1:** General characteristics of study objects (n = 200).

	Total	Valgus deformity	Valgus deformity & LMLA	*P* value
N	200	26 (13.0%)	174 (87.0%)	
Gender				
Male	62 (31.0%)	9 (34.6%)	53 (30.5%)	.669
Female	138 (69.0%)	17 (65.4%)	121 (69.5%)	
Age	6.49 ± 2.96	7.08 ± 3.26	6.40 ± 2.91	.675

Data presented as mean ± SD.

LMLA = lowered of a medial longitudinal arch.

As the angles were measured from radiographs, CPA and TMA showed significant improvement (*P* < .001). However, CPA in the right foot of the group with valgus deformity was the only exception (*P* = .078) (Table 2). In addition, to confirm the correlation of treatment effect according to age, an analysis was performed based on the age of 6 years. Both CPA and TMA also showed significant improvements regardless of the age group, including preschool age (6 ≥ age) and school-age (6 < age) (*P* < .001) (Table 3).

**Table 2:** Comparison between calcaneal pitch angle (CPA) and talus-first metatarsal angle (TMA) after pre- and post- foot insole application in flat foot subjects.

	n	CPA									TMA							
		Right					Left				Right				Left			
		Pre	Post	Δ (Post-Pre)	*P* value	Pre		Post	Δ (Post-Pre)	*P* value	Pre	Post	Δ (Post-Pre)	*P* value	Pre	Post	Δ (Post-Pre)	*P* value
Total	200	11.22 ± 4.73	14.51 ± 3.84	3.30 ± 3.23	<.001*	11.20 ± 5.08		14.48 ± 3.93	3.28 ± 3.39	<.001*,†	20.94 ± 8.24	13.11 ± 7.78	−7.83 ± 7.74	<.001*	20.04 ± 7.48	14.26 ± 8.27	−5.78 ± 7.51	<.001*
Valgus deformity	26	18.83 ± 2.15	19.79 ± 2.23	0.96 ± 2.66	.078	17.99 ± 2.57		19.85 ± 2.05	1.86 ± 2.50	<.002*	16.90 ± 5.93	11.62 ± 6.03	−5.28 ± 6.50	<.001*	17.35 ± 5.14	11.24 ± 6.67	−6.11 ± 7.91	.001*
Valgus deformity & LMLA	174	10.08 ± 3.88	13.72 ± 3.38	3.65 ± 3.17	<.001*	10.19 ± 4.56		13.68 ± 3.49	3.49 ± 3.46	<.003*,†	21.55 ± 8.38	13.33 ± 7.99	−8.22 ± 7.85	<.001*	20.44 ± 7.70	14.71 ± 8.41	−5.73 ± 7.47	<.001*

Data presented as mean ± SD.

CPA = calcaneal pitch angle, LMLA = lowered of a medial longitudinal arch, TMA = talo first metatarsal angle.

* *P* < .05.

† Wilcoxon signed-rank test.

**Table 3:** Comparison between different age groups in flat foot subjects.

	n	CPA								TMA							
		Right				Left				Right				Left			
		Pre	Post	Δ (Post-Pre)	*P* value	Pre	Post	Δ (Post-Pre)	*P* value	Pre	Post	Δ (Post-Pre)	*P* value	Pre	Post	Δ (Post-Pre)	*P* value
Age ≤ 6	110	11.05 ± 4.69	14.77 ± 3.63	3.72 ± 3.54	<.001*	10.63 ± 4.59	14.34 ± 3.75	3.71 ± 3.05	<.001*,†	21.64 ± 8.37	13.19 ± 7.74	−8.45 ± 7.71	<.001*	21.15 ± 7.23	14.50 ± 8.34	−6.64 ± 7.54	<.001†,*
Valgus deformity	12	18.78 ± 2.40	19.87 ± 2.51	1.09 ± 2.98	.233	18.01 ± 1.41	19.88 ± 2.54	1.87 ± 2.44	.022*	15.78 ± 6.49	10.99 ± 6.06	−4.79 ± 6.75	.032*	17.12 ± 5.41	10.38 ± 7.56	−6.74 ± 8.24	.016*
Valgus deformity & LMLA	98	10.10 ± 3.98	14.14 ± 3.23	4.04 ± 3.48	<.001*	9.73 ± 3.99	13.66 ± 3.29	3.93 ± 3.06	<.001*,†	22.35 ± 8.32	13.46 ± 7.91	−8.89 ± 7.73	<.001*	21.64 ± 7.29	15.01 ± 8.33	−6.63 ± 7.50	<.001†*
Age > 6	90	11.42 ± 4.78	14.20 ± 4.08	2.78 ± 2.74	<.001*	11.90 ± 5.57	14.66 ± 4.16	2.76 ± 3.70	<.001*,†	20.09 ± 8.05	13.01 ± 7.86	−7.09 ± 7.75	<.001*	18.68 ± 7.59	13.96 ± 8.23	−4.72 ± 7.37	<.001*
Valgus deformity	14	18.87 ± 2.00	19.72 ± 2.04	0.85 ± 2.45	.219	17.98 ± 3.31	19.83 ± 1.62	1.84 ± 2.64	.022*	17.86 ± 5.46	12.16 ± 6.17	−5.70 ± 6.51	.006*	17.54 ± 5.09	11.97 ± 5.99	−5.57 ± 7.89	.020*
Valgus deformity & LMLA	76	10.05 ± 3.77	13.18 ± 3.50	3.13 ± 2.65	<.001*	10.78 ± 5.17	13.71 ± 3.77	2.93 ± 3.86	<.001*,†	20.50 ± 8.40	13.16 ± 8.16	−7.34 ± 7.97	<.001*	18.89 ± 7.97	14.33 ± 8.56	−4.56 ± 7.32	<.001*

Data presented as mean ± SD.

CPA = calcaneal pitch angle, LMLA = lowered of a medial longitudinal arch, TMA = talo first metatarsal angle.

* *P* < .05.

† Wilcoxon signed-rank test.

## 4. Discussion

Several studies have been conducted using foot orthosis as a conservative treatment. Kuhn et al^[15]^ demonstrated that custom made flexible orthoses instantly improved the alignment of the pedal structure measured using radiological methods. Sinha et al^[16]^ found that medial arch support orthoses not only improved the American Orthopedic Foot and Ankle Society scores for pain, but also improved foot angles, especially in the CPA and lateral talocalcaneal angles. Some studies have reported that short-term insole application also improves pain, comfort, and balancing ability.^[17,18]^ At the 1-year follow up study, the signs of flatfoot were reduced by wearing insoles in preschool aged children (3 to 5 years old) according to the Chippaus–Smirak index.^[19]^ Several studies have performed long term follow up for a minimum of 2 years to a maximum of 6 years using custom-made rigid foot orthoses, which revealed improvements in radiologic indices, such as the talometatarsal, metatarsal, and calcaneal pitch angles.^[20–22]^ According to the results of sequentially conducted studies, the effect of orthopedic braces was proven; however, it was limited to patients of preschool age, the number of subjects was small, or there were limitations in using evaluation tools other than radiographs.

In this study, we gathered the medical records of children diagnosed with SFFF. They were followed up every 3 to 4 months by wearing custom made foot insoles, which were revised after radiological confirmation. The termination of treatment was determined by the disappearance of symptoms, improvement of radiologic indices, and duration, which was minimal at 1 year and maximum at 6 years. The analytical results showed a significant improvement in individual radiologic values. Furthermore, age groups, including the preschool age (6 years ≥ age) and school age groups (6 years < age) showed enhancement, except for the group with valgus deformity in the right foot. Above all, this study judged the effect through radiographs by classifying them into preschool age and post school age groups, and the number of subjects was sufficiently large.

Despite many studies showing advancement, as stated above, including our study, contrasting results could also be found. Wenger et al^[23]^ randomly assigned patients with typical flexible flatfoot into 4 groups. They followed up children for 3 years with great care to provide proper shoe fit. The results showed that there were no significant differences between the groups; therefore, the author did not recommend corrective shoes or inserts. However, this study was conducted in a limited age range. Additionally, the degree of the subject with flexible flatfoot was relatively mild. Similarly, Kanatli et al^[24]^ researched the effect of orthopedic shoes that could change the development of foot arches. The study showed no noticeable changes in the corrective shoe wearing group and the no treatment group; however, the enrolled groups were only preschool children with a mean age of 39.5 months and were pain free.

One study reported a randomized controlled trial of 2 types of in shoe orthosis, which were custom made, ready-made, and control groups.^[25]^ They used calcaneal eversion and navicular drop as diagnostic tools, and most outcome measures were statistically significant, but not in comparisons among the groups. The reason is that the commonly used measurement was weakly correlated, which made them difficult to interpret, and the authors did not conduct evaluations using radiologic assessment.

Riccio et al^[26]^ attempted to prove the effectiveness of a rehabilitative program for maintaining foot flexibility. The study finally insisted that using a foot orthosis is no longer opportune because the program produced significantly better results, but showed a limitation in the selection of subjects, such as age (mean age: 3.4 years), inclusion of a valgus form of flatfoot only, and information on whether symptoms were present.

Contrary to the above research, the present study showed that significant outcomes in the clinical symptoms and radiographic improvement were observed when appropriate insoles were worn in symptomatic children with flat feet, regardless of age.

There are limitations to the current study and some points to be supplemented. First, this was a retrospective study. The selection of control groups with a sufficient treatment duration should be performed in future research. Second, the consideration of the role of parents was insufficient. Most studies on children require the presence of parents. Their participation has a significant impact on the research resultsin terms of objectivity. For example, the parents of some children voluntarily stopped treatment. The reasons for their discontinuation were their convenience, difficulty in observing their child’s uncomfortable appearance, and the fact that they thought the treatment had been performed without any medical or objective basis. Although the results of our study showed clinical improvement leading to the end of treatment, we cannot exclude the possibility that parental intervention was completely blocked. If additional systematic protocols are provided to ensure objectivity, changes in treatment duration and radiologic values could be expected. Moreover, we focused only on the limited variables, results, and changed values. Factors, such as weight, height, hypermobility of joints, and laxity in ligaments are already known to be important conditions. Further studies are needed not only to apply these factors, but also to determine the age or duration of treatment that is most effective.

## 5. Conclusion

In this study, a long term but periodically revised foot insole significantly improved radiological indicators, both CPA and TMA, in children diagnosed with SFFF under 18 years of age. When the angles were measured from the radiographs, both angles showed significant improvement. Therefore, we suggest that foot insoles are an effective treatment modality for symptomatic pediatric flatfoot.

## Acknowledgments

We would like to thank Nam Hun Heo from the clinical trial center of Soonchunhyang University Cheonan Hospital for advice on statistical analysis.

## Author contributions

**Conceptualization:** Soo A Kim, Yuntae Kim.

**Formal analysis:** Nam Hun Heo.

**Writing – original draft:** Joon Yeop Kim, Soo A Kim.

**Writing – review & editing:** Soo A Kim, Insu Hwang.
